# Chemokine gene expression in lung CD8 T cells correlates with protective immunity in mice immunized intra-nasally with Adenovirus-85A

**DOI:** 10.1186/1755-8794-3-46

**Published:** 2010-10-13

**Authors:** Lian N Lee, Dilair Baban, Edward O Ronan, Jiannis Ragoussis, Peter CL Beverley, Elma Z Tchilian

**Affiliations:** 1 Nuffield Department of Medicine, University of Oxford, The Peter Medawar Building for Pathogen Research, South Parks Road, Oxford OX1 3SY, UK; 2 The Wellcome Trust Centre for Human Genetics, University of Oxford, Roosevelt Drive, Oxford OX3 7BN, UK

## Abstract

**Background:**

Immunization of BALB/c mice with a recombinant adenovirus expressing *Mycobacterium tuberculosis *(*M. tuberculosis*) antigen 85A (Ad85A) protects against aerosol challenge with *M. tuberculosis *only when it is administered intra-nasally (i.n.). Immunization with Ad85A induces a lung-resident population of activated CD8 T cells that is antigen dependent, highly activated and mediates protection by early inhibition of *M. tuberculosis *growth. In order to determine why the i.n. route is so effective compared to parenteral immunization, we used microarray analysis to compare gene expression profiles of pulmonary and splenic CD8 T cells after i.n. or intra-dermal (i.d.) immunization.

**Method:**

Total RNA from CD8 T cells was isolated from lungs or spleens of mice immunized with Ad85A by the i.n. or i.d. route. The gene profiles generated from each condition were compared. Statistically significant (p ≤ 0.05) differentially expressed genes were analyzed to determine if they mapped to particular molecular functions, biological processes or pathways using Gene Ontology and Panther DB mapping tools.

**Results:**

CD8 T cells from lungs of i.n. immunized mice expressed a large number of chemokines chemotactic for resting and activated T cells as well as activation and survival genes. Lung lymphocytes from i.n. immunized mice also express the chemokine receptor gene *Cxcr6*, which is thought to aid long-term retention of antigen-responding T cells in the lungs. Expression of CXCR6 on CD8 T cells was confirmed by flow cytometry.

**Conclusions:**

Our microarray analysis represents the first *ex vivo *study comparing gene expression profiles of CD8 T cells isolated from distinct sites after immunization with an adenoviral vector by different routes. It confirms earlier phenotypic data indicating that lung i.n. cells are more activated than lung i.d. CD8 T cells. The sustained expression of chemokines and activation genes enables CD8 T cells to remain in the lungs for extended periods after i.n. immunization. This may account for the early inhibition of *M. tuberculosis *growth observed in Ad85A i.n. immunized mice and explain the effectiveness of i.n. compared to parenteral immunization with this viral vector.

## Background

It is becoming increasingly apparent that when T cells are essential for protective immunity the route of vaccine delivery may be critical [1-4]. Mice immunized intra-dermally (i.d.) or intra-muscularly (i.m.) with recombinant adenovirus expressing *Mycobacterium tuberculosis *(*M. tuberculosis*) antigen 85A (Ad85A) make a very strong splenic CD8 T cell response, but show no reduction in the lung mycobacterial burden after pulmonary challenge with *M. tuberculosis *compared to naïve mice. In contrast, mice immunized intra-nasally (i.n.) with Ad85A make much weaker splenic responses but develop a very strong lung CD8 T cell response to antigen 85A and are able to reduce significantly the mycobacterial burden after *M. tuberculosis *challenge [2,5,6]. Regardless of the route of delivery, immunization with Ad85A generates a predominantly CD8 T cell response in BALB/c mice, characterized by production of IFNγ, TNF and some IL-2. We and others have shown that in this model the localization and continued presence of 85A-specific cells at the site of pathogen entry is dependent on the presence of antigen in the lungs and correlates with protection [5,7,8]. Furthermore, *M. tuberculosis *growth in the lungs is inhibited during the first 8 days after infection in Ad85A i.n. immunized mice. This is in contrast to mice immunized parenterally with BCG, in which the kinetics of *M. tuberculosis *growth are unchanged compared to naïve mice up to day 14 [5]. Thus it appears that the presence of activated effector T cells in the lungs plays a role in the protective immunity induced by Ad85A i.n. immunization. Nevertheless, these data do not satisfactorily explain why mice immunized i.d., which make a strong systemic immune response to 85A, fail to show any protection against pulmonary *M. tuberculosis *infection or dissemination of *M. tuberculosis *to the spleen.

We therefore sought to determine whether there are any differences in gene expression in the CD8 T cells induced by Ad85A i.d. or i.n. immunization that might explain the difference in protection. Microarray analysis was performed on RNA from lung and spleen CD8 T cells from Ad85A i.n. or i.d. immunized mice. The transcriptional profiles of lung CD8 T cells from Ad85A i.n. immunized mice show higher expression of chemokines, activation markers and tissue homing receptors, which may enable them to reside in the lung for extended periods.

## Methods

### Animals and immunization

All experiments were performed with 6-8 week old female BALB/c mice (Harlan Orlac, Blackthorn, UK), were approved by the animal use ethical committee of Oxford University and fully complied with the relevant Home Office guidelines. Mice were immunized with a recombinant replication deficient adenovirus serotype 5 containing the 85A antigen from *M. tuberculosis *(Ad85A) [5]. For intra-dermal (i.d.) immunization mice were anaesthetized and injected with 25 μl in each ear, containing a total of 2 × 10^9 ^virus particles (v.p.) of Ad85A per mouse and for i.n. immunization allowed slowly to inhale 50 μl of 2 × 10^9 ^v.p. of Ad85A.

### Enrichment of CD8 T cells from lung and spleen and RNA extraction

Lungs were perfused with PBS, cut into small pieces and digested with 0.7 mg/ml collagenase type I (Sigma, Poole, UK) and 30 μg/ml DNase I (Sigma) for 45 min at 37°C. Lung fragments were then crushed through a cell strainer using a 5 ml syringe plunger, washed, layered over Lympholyte (Cederlane, Ontario, Canada) and centrifuged. Interface cells were washed and CD8 T cells selected by adding CD8 Microbeads (Miltenyi Biotec, Bisley, UK), followed by positive selection on MACS columns. The isolated cells were placed in Trizol and total RNA extracted using chloroform and isopropanol followed by cleanup on Qiagen RNeasy MinElute spin columns (Qiagen, Crawley, UK). RNA was quantitated and purity was determined on a Nanodrop (Thermo Scientific, Loughborough, UK). The spleens were passed through a cell strainer using a 5 ml syringe plunger, then red blood cells were lysed using RBC lysis buffer (Qiagen). The cells were washed and CD8 T cells positively selected using CD8 Microbeads, followed by extraction of total RNA as described for the lung samples. For each immunization route separate pools of 7 lungs or spleens were made and subjected to microarray analysis.

### Microarray analysis

Genome-wide gene expression analysis was performed using MouseWG6_v2 beadchips (Illumina, Little Chesterford, UK) containing 45,200 annotated transcripts. Briefly, total RNA was amplified and labeled with biotin during *in vitro *transcription, then hybridized to the array, washed, stained with Cy3-Streptavidin complex and subsequently scanned. QC and gene detection was performed using GenomeStudio (Illumina). For each condition, RNA samples obtained from 3 independent experiments were tested separately to generate triplicate sets of data per condition. The data files are available at the Gene Expression Omnibus data repository: GSE23713. The genes detected from each condition were compared against others to determine statistically significant (p ≤ 0.05) fold changes in expression. The lists of differentially expressed genes and their corresponding fold change values were subsequently analyzed using Gene Ontology [9] and Panther DB websites [10] to determine whether the changes mapped to particular molecular functions, biological processes or pathways.

### Aerosol *M. tuberculosis *challenge

Four weeks after the Ad85A immunization, mice were challenged by aerosol with *M. tuberculosis *(Erdman strain, kindly provided by Dr Amy Yang, CBER/FDA) using a modified Henderson apparatus [11]. Deposition in the lungs was measured 24 h after *M. tuberculosis *challenge and was ~ 200 CFU per lung. Mice were sacrificed at 6 weeks after *M. tuberculosis *challenge. Spleens and lungs were homogenized and the bacterial load was determined by plating 10-fold serial dilutions of tissue homogenates on Middlebrook 7H11 agar plates (E & O Laboratories Ltd, Bonnybridge, UK). Colonies were counted after 3-4 weeks of incubation at 37°C in 5% CO_2_.

### Flow cytometry

Lung cells were cultured in DMEM supplemented with 10% heat-inactivated FBS, L-glutamine, penicillin and streptomycin. Cells were stimulated with a mix of 3 peptides (Peptide Protein Research Ltd, Fareham, UK) encoding dominant and subdominant CD8 and dominant CD4 epitopes of antigen 85A [5]. Each peptide was at a final concentration of 2 μg/ml during the stimulation. After 1 hour at 37°C Golgi Plug (BD Biosciences, Oxford, UK) was added according to the manufacturer's instructions and cells were incubated for an additional 5 hours before intracellular cytokine staining.

Cells were washed and incubated with CD16/CD32 mAB to block Fc binding. Subsequently the cells were stained for CD4 (RM4-5), IFNγ (XMG1.2), IL-2 (JES6-5H4), TNF (MP6-XT22), Lag3 (C9B7W) and Ly 6A (D7) (eBioscience, Hatfield, UK), CXCR6 antibody (221002) (R&D Systems, Abingdon, UK), and CD8 (53-6.7) (BD Bioscience). For intracellular cytokine staining, cells were stained using the BD Cytofix/Cytoperm kit according to the manufacturer's instructions. Cells were fixed with PBS 1% paraformaldehyde, run on a LSRII (BD Biosciences) and analyzed using FlowJo software (Tree Star Inc, Ashland, Oregon, USA).

### Detection of chemokines by ELISA

Lung lymphocytes were isolated as described above and incubated in RPMI+10% FCS at 37°C for 6 hours without stimulation. The supernatant was collected and stored at -80°C until analysis. CXCL16 (R&D Systems) ELISA and Multi-Analyte ELISArray for Mouse Common Chemokines (SABiosciences, Frederick, MD, USA) kits were employed.

## Results

### Immunization with Ad85A i.n. or i.d

**Figure 1:**
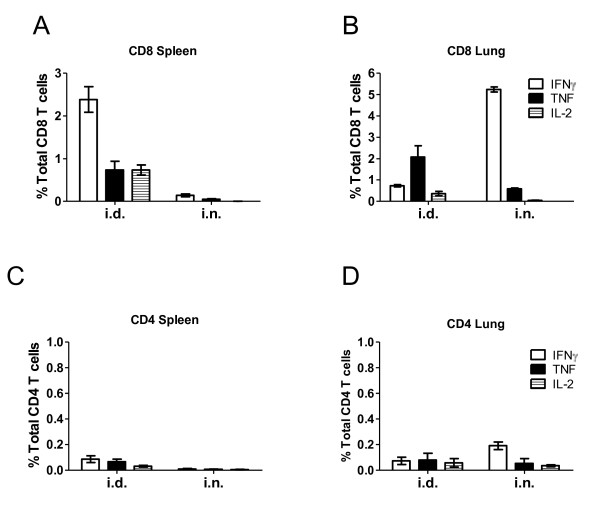
**Cytokine responses of T cells to antigen 85A**. BALB/c mice were immunized with Ad85A i.d. or i.n.. Lung and splenic lymphocytes were isolated 3 weeks post-immunization and stimulated with the dominant CD4 and dominant and subdominant CD8 peptides. The percentage of cells expressing IFNγ, TNF and IL-2 in (A) splenic CD8, (B) lung CD8, (C) splenic CD4 and (D) lung CD4 T cells as determined by flow cytometry. The values shown are the mean ± SEM from 3 mice per group and are representative of results obtained from at least 2 independent experiments.

**Figure 2:**
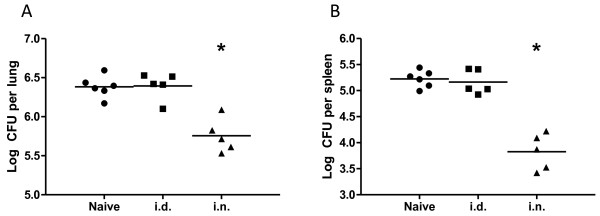
**Control of mycobacterial growth after aerosol *M. tuberculosis *challenge of Ad85A immunized mice**. BALB/c mice were immunized with Ad85A i.d. or i.n. and challenged 4 weeks later with *M. tuberculosis *by aerosol. Mice were sacrificed 6 weeks later and mycobacterial burden in the lungs (A) and spleen (B) determined. The results show the log CFU in each mouse and the mean for each group and are representative of at least 2 independent experiments. The data were analyzed using the Kruskal-Wallis test (p = 0.007 comparing all groups), followed by Dunn's multiple comparison test, which returned p-values of < 0.05 for comparisons between naïve vs. i.n. and i.d. vs. i.n. groups. * indicates p < 0.05 compared to naïve or i.d.

In agreement with previous reports [2,5,6], mice immunized with Ad85A i.n. developed a strong 85A-specific T cell response in the lung and a much weaker splenic response, while Ad85A i.d. immunized mice make a stronger spleen and weaker lung response (Figure 1). Ad85A i.n. mice reduced the lung mycobacterial load by ~ 1 log compared to naïve mice (Figure 2A), a protective effect comparable to BCG [5,12], while Ad85A i.d. immunization did not reduce the bacterial load either in the lungs or spleen (Figure 2B). The responses induced by Ad85A in BALB/c mice are dominated by CD8 T cells (Figure 1). In C57BL/6 mice Ad85A immunization induces a CD4 response and no consistent statistically significant protection against pulmonary *M. tuberculosis *challenge is obtained. However in BALB/c mice it has been demonstrated that antigen-specific CD8 T cells in the lung are critical for protection against pulmonary infection with *M. tuberculosis *in this immunization model [7,13]. Therefore we wished to determine if CD8 T cells from the lungs and spleens of BALB/c mice differed. Mice were immunized i.n. or i.d. with Ad85A and CD8 T cells isolated from the lungs (lung i.n. or i.d.) or spleens (spleen i.n. or i.d.) 3 weeks post-immunization, close to the peak of the cytokine response [5]. Purification by positive selection resulted in CD8 T cell populations which were 80-86% pure from lungs and 86-90% pure from spleens. Total RNA was prepared from cells isolated in 3 independent experiments, amplified and global genome analysis was performed using Illumina microarrays. The expression profiles of spleen and lung CD8 T cells were compared. Transcripts with ≥ 2 fold difference in signal intensity with a p-value of ≤ 0.05 were considered for further analysis, apart from the comparison between spleen i.n. and spleen i.d. where transcripts with ≥ 1.5 fold difference were considered.

### Comparison of gene expression between lung i.n. and spleen i.d

**Figure 3:**
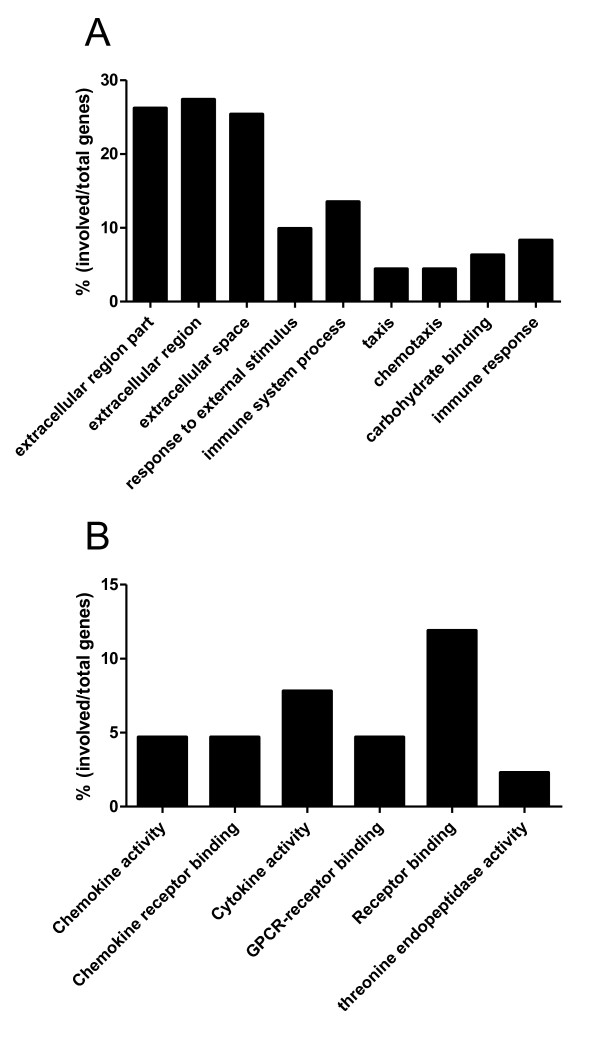
**Gene Ontology analysis of differentially expressed genes in CD8 T cells**. (A) Spleen i.d. vs lung i.n., based on 550 differentially expressed transcripts and (B) lung i.n. vs lung i.d. based on 245 differentially expressed transcripts (>2 fold difference, p < 0.05).

We compared the expression profiles of lung i.n. (protective regime) with spleen i.d. (non-protective regime) CD8 T cells. 550 transcripts were found to be differentially expressed (Additional file 1), with 186 transcripts more highly expressed by the lung i.n. sample and 364 transcripts by the spleen i.d. samples. Gene Ontology mapping of the 550 differentially expressed genes indicated that a large proportion were related to expression of extracellular proteins or involved in responses to extracellular stimuli or immune system processes (Figure 3A).

In agreement with this, when the list of differentially expressed genes was classified according to function using Panther analysis, 105 of the 550 differentially expressed transcripts were found to be involved in immune-related processes (Table 1). Of these 105 transcripts, 64 were more highly expressed in spleen i.d. samples, many of which were classified as related to either inflammation, complement- or ligand-mediated signaling processes, signal transduction or other host immune responses (Table 1). Additionally, higher levels of transcripts for antimicrobial molecules such as cathelicidin (*Camp*) (section XI Table 1) and lactotransferrin (*Ltf*) (section XII Table 1) were detected. Human cathelicidin has been reported to inactivate adenoviruses [14] and lactotransferrin to mediate entry of adenovirus subtype 5 virus into cells [15]. The only chemokine-related genes more highly expressed in spleen i.d. samples were *Ccl24*, along with its cognate receptor *Ccr3*. In addition to processes associated with immunity, the Panther program also predicted that many genes more highly expressed in spleen i.d. samples play a role in numerous and diverse non-immune associated processes such as blood circulation and gas exchange, blood clotting, porphyrin metabolism, sensory perception, proteolysis and cell structure and motility (data not shown).

**Table 1:** Differentially expressed host genes which mapped to immune related responses.

Annotation	Name	Accession	Fold Difference	
**I Cytokine and chemokine-mediated immunity and signalling**			**lung i.n. vs spleen i.d**.	**lung i.n.vs lung i.d**.

CD40 antigen	*Cd40*	NM_170702.2		3.0

Chemokine (C motif) ligand 1	*Xcl1*	NM_008510.1	4.0	8.0

Chemokine (C-C motif) ligand 1	*Ccl1*	NM_011329.1	9.7	6.7

Chemokine (C-C motif) ligand 2	*Ccl2*	NM_011333.1	3.2	2.2

Chemokine (C-C motif) ligand 3	*Ccl3*	NM_011337.1		4.3

Chemokine (C-C motif) ligand 4	*Ccl4*	NM_013652.1		7.4

Chemokine (C-C motif) ligand 5	*Ccl5*	NM_013653.1		3.4

Chemokine (C-C motif) ligand 7	*Ccl7*	NM_013654	17.9	6.9

Chemokine (C-C motif) ligand 8	*Ccl8*	NM_021443.1	10.7	4.3

Chemokine (C-C motif) ligand 12	*Ccl12*	NM_011331	2.7	

Chemokine (C-C motif) ligand 24	*Ccl24*	NM_019577.2	-4.3	

Chemokine (C-X-C motif) ligand 1	*Cxcl1*	NM_008176.1	2.9	

Chemokine (C-X-C motif) ligand 2	*Cxcl2*	NM_009140	2.4	

Chemokine (C-X-C motif) ligand 9	*Cxcl9*	NM_008599		12.6

Chemokine (C-C motif) receptor 3	*Ccr3*	NM_009914.2	-2.1	

Chemokine (C-X-C motif) receptor 6	*Cxcr6*	NM_030712.1		5.3

Colony stimulating factor 1 (macrophage)	*Csf1*	NM_007778.1	2.1	

Colony stimulating factor 3 receptor (granulocyte)	*Csf3r*	NM_007782.1	-2.1	

Cytokine inducible SH2-containing protein	*Cish*	NM_009895.2	4.7	4.5

Interferon gamma	*Ifng*	NM_008337.1		5.2

Interferon gamma receptor 2	*Ifngr2*	NM_008338.2	3.2	

Interleukin 18	*Il18*	NM_008360.1	-4.3	

Lymphocyte-activation gene 3	*Lag3*	NM_008479.1	2.8	5.0

Protein tyrosine phosphatase, non-receptor type 6	*Hcph*	NM_013545.1		2.0

Signal-regulatory protein alpha	*Ptp4a3*	NM_008975.2	-2.1	

Transferrin	*Trf*	NM_133977.1	-8.8	

Tumor necrosis factor	*Tnf*	NM_013693		2.6

				

**II Inflammation mediated pathway**				

Cyclin-dependent kinase inhibitor 1A (P21)	*Cdkn1a*	NM_007669.2	2.2	

Gardner-Rasheed feline sarcoma viral (Fgr) oncogene homolog	*Fgr*	NM_010208	-3.5	

Integrin alpha 9	*Itga9*	NM_133721.1	-3.0	

Procollagen, type XIV, alpha 1	*Col14a1*	AK052963	-3.4	

Prostaglandin-endoperoxide synthase 1	*Ptgs1*	NM_008969.1	-7.0	

Vav2 oncogene	*Vav2*	NM_009500.1	-3.7	

				

				

**III Interferon-stimulated genes**				

2'-5' oligoadenylate synthetase-like 2	*Oasl2*	NM_011854.1	4.0	3.3

2'-5' oligoadenylate synthetase 1G	*Oas1g*	NM_011852.2		3.2

Guanylate nucleotide binding protein 1	*Gbp1*	NM_010259.1	2.2	5.5

Guanylate nucleotide binding protein 2	*Gbp2*	NM_010260.1		4.4

Guanylate nucleotide binding protein 4	*Gbp4*	NM_018734.2		3.2

Interferon activated gene 202B	*Ifi202b*	NM_008327.1		2.4

Interferon-induced protein with tetratricopeptide repeats 2	*Ifit2*	NM_008332.2		2.0

Interferon-induced protein with tetratricopeptide repeats 3	*Ifit3*	NM_010501.1		3.9

Interferon regulatory factor 8	*Icsbp1*	NM_008320.2		3.2

Signal transducer and activator of transcription 1	*Stat1*	NM_009283		3.0

SLAM family member 8	*Slamf8*	XM_129596.2		5.0

				

**IV T cell activation**				

Beta-2 microglobulin	*B2m*	NM_009735.2		2.3

Cathepsin S	*Ctss*	NM_021281.1		3.0

CD74 antigen (invariant polypeptide of major histocompatibility complex, class II antigen-associated)	*li*	BC003476		5.6

CD86 antigen	*Cd86*	NM_019388.2	-2.3	2.5

CD274 antigen	*Pdcd1lg1*	NM_021893.2	2.5	3.8

Cytotoxic T-lymphocyte-associated protein 4	*Ctla4*	NM_009843.2		3.9

Histocompatibility 2, Q region locus 6	*H2-Q6*	NM_207648		3.0

Interferon gamma inducible protein 30	*Ifi30*	NM_023065.2		2.5

Histocompatibility 2, class II antigen A, beta 1	*H2-Ab1*	NM_207105.1		7.7

Histocompatibility 2, D region locus 1	*H2-D1*	NM_010380.2		3.0

Histocompatibility 2, class II, locus Dma	*H2-DMa*	NM_010386		3.9

Histocompatibility 2, class II, locus Mb1	*H2-DMb1*	NM_010387.2		5.8

Histocompatibility 2, class II, locus Mb2	*H2-DMb2*	NM_010388		5.5

Histocompatibility 2, class II antigen E alpha	*H2-Ea*	NM_010381.2		6.5

Histocompatibility 2, class II antigen E beta	*H2-Eb1*	NM_010382.1		7.9

Histocompatibility 2, K1, K region	*H2-K1*	NM_001001892.1		3.0

Histocompatibility 2, M region locus 3	*H2-M3*	NM_013819.1		2.6

Histocompatibility 2, Q region locus 2	*H2-Q2*	NM_010392.2		2.7

Histocompatibility 2, Q region locus 5	*H2-Q5*	NM_010393.1		2.9

Histocompatibility 2, T region locus 10	*H2-T10*	NM_010395.5		2.5

Histocompatibility 2, T region locus 23	*H2-T23*	NM_010398.1		2.9

Histocompatibility 2, T region locus 9	*H2-T9*	NM_010399		3.1

Peroxiredoxin 5	*Prdx5*	NM_012021		2.2

				

**V Other ligand-mediated signalling**				

Calcitonin/calcitonin-related polypeptide, alpha	*Calca*	NM_007587	2.0	

E2F transcription factor 2	*E2f2*	NM_177733.2	-3.4	

Endothelin 1	*Edn1*	NM_010104.2	2.1	

Endothelial differentiation, sphingolipid G-protein-coupled receptor, 5	*Edg5*	NM_010333		2.1

FMS-like tyrosine kinase 1	*Flt1*	NM_010228.2	3.9	

Granulin	*Grn*	NM_008175.2		2.8

Mannose-6-phosphate receptor, cation dependent	*M6pr*	NM_010749.4		2.2

Paired-Ig-like receptor A1	*Pira1*	NM_011087.1	-2.1	

Paired-Ig-like receptor A11	*Pira11*	NM_011088.1	-2.1	

Paired-Ig-like receptor A3	*Pira3*	NM_011090.1	-3.8	

Paired-Ig-like receptor A4	*Pira4*	NM_011091.1	-2.4	

Peroxisome proliferator activated receptor gamma	*Pparg*	NM_011146.1	-2.7	

Pre-B-cell colony-enhancing factor 1	*Pbef1*	NM_021524.1		2.7

Secretoglobin, family 1A, member 1 (uteroglobin)	*Scgbla1*	NM_011681.1	40.0	

Solute carrier family 1 (glial high affinity glutamate transporter), member 3	*Slcla3*	NM_148938.2	-2.5	

Spermine oxidase	*Smox*	NM_145533.1	-2.7	

Vascular endothelial growth factor A	*Vegfa*	NM_009505.2	2.5	

				

**VI Complement-mediated**				

Complement component 1, q subcomponent, alpha polypeptide	*C1qa*	NM_007572	-3.6	

Complement component 1, q subcomponent, beta polypeptide	*C1qb*	NM_009777.1	-4.5	3.8

Complement component 1, q subcomponent, C chain	*C1qg*	NM_007574.1	-4.3	

Complement component 2 (within H-2S)	*C2*	NM_013484.1	-3.3	

Complement component 3	*C3*	NM_009778.1		4.2

Complement component 4A (Rodgers blood group)	*Slp*	NM_011413	2.3	

Complement component 6	*C6*	NM_016704.1	-2.6	

Complement factor B	*H2-Bf*	NM_008198.1	11.5	6.5

Complement factor properdin	*Pfc*	XM_135820.3	-10.3	

Four and a half LIM domains 1	*Fhl1*	NM_010211.1	2.3	

				

**VII Apoptosis**				

B-cell leukemia/lymphoma 2 related protein A1b	*Bcl2a1b*	NM_007534		2.5

B-cell leukemia/lymphoma 2 related protein A1d	*Bcl2a1d*	NM_007536		2.7

Caspase 1	*Casp1*	NM_009807.1		4.0

Caspase 4, apoptosis-related cysteine peptidase	*Casp4*	NM_007609.1		2.4

Epithelial membrane protein 3	*Emp3*	NM_010129.1		2.3

Lectin, galactose binding, soluble 1	*Lgals1*	NM_008495.1		4.0

Thioredoxin 1	*Txn1*	NM_011660.3		2.1

				

**VIII Signal transduction**				

AXL receptor tyrosine kinase	*Axl*	NM_009465.2	-6.4	

C-mer proto-oncogene tyrosine kinase	*Mertk*	NM_008587	-3.7	

C-type lectin domain family 4, member a1	*BC049354*	XM_194289.2	-3.0	

C-type lectin domain family 4, member b1	*Clec4b1*	NM_027218.1	-3.9	

C-type lectin domain family 4, member a3	*Clec4a3*	NM_153197.3	-2.1	

CD63 antigen	*Cd63*	NM_007653.1	3.3	

CD81 antigen	*Cd81*	NM_133655.1	-4.6	

CD93 antigen	*C1qr1*	NM_010740.1	3.7	

CD207 antigen	*Cd207*	NM_144943.2	-2.2	

Chemokine (C-X-C motif) ligand 15	*Cxcl15*	NM_011339.1	5.0	

Colony stimulating factor 1 receptor	*Csf1r*	NM_007779.1	-9.5	

Integrin beta 5	*Itgb5*	NM_010580	-3.4	

Neutrophilic granule protein	*Ngp*	NM_008694.1	-8.9	

Plasminogen activator, urokinase	*Plau*	NM_008873.2	2.2	

S100 calcium binding protein A9 (calgranulin B)	*S100a9*	NM_009114.1	-4.2	

Thrombomodulin	*Thbd*	NM_009378.1	4.6	

				

**IX Other cell communication**				

Cadherin 1	*Cdh1*	NM_009864.1	4.8	3.2

Hematological and neurological expressed sequence 1	*Hn1*	NM_008258.1		2.3

Intercellular adhesion molecule	*Icam1*	NM_010493.2		2.3

Lectin, galactoside-binding, soluble, 3 binding protein	*Lgals3bp*	NM_011150.1		4.0

Proteolipid protein 2	*Plp2*	NM_019755.2		2.5

Purine-nucleoside phosphorylase	*Pnp*	NM_013632.2		3.2

				

**X Natural killer cell-mediated immunity**				

Fc fragment of IgG, low affinity IIIa, receptor	*Fcrl3*	NM_144559.1		3.8

Killer cell lectin-like receptor subfamily C, member 1	*Klrc1*	NM_010652		3.8

Killer cell lectin-like receptor subfamily K, member 1	*Klrk1*	NM_033078.2		2.0

Natural killer cell group 7 sequence	*Nkg7*	NM_024253.3		3.7

				

**XI Other host immune responses**				

Allograft inflammatory factor 1	*Aif1*	NM_019467.2	-2.8	2.7

ATP-binding cassette, sub-family C (CFTR/MRP), member 3	*Abcc3*	XM_358306.1	-10.6	

Carboxylesterase 3	*Ces3*	NM_053200.1	2.9	

Catalase	*Cat*	NM_009804.1	-2.3	

Cathelicidin antimicrobial peptide	*Camp*	NM_009921.1	-18.3	

Cathepsin E	*Ctse*	NM_007799	-2.3	

CD59a antigen	*Cd59a*	NM_007652.2	-3.0	

CD93 antigen	*C1qr1*	NM_010740.1	3.7	

CD163 antigen	*Cd163*	NM_053094.1	-10.1	

CD244 natural killer cell receptor 2B4	*Cd244*	NM_018729	-3.3	

Coagulation factor X	*F10*	NM_007972.2	2.6	

Fc receptor, IgG, alpha chain transporter	*Fcgrt*	NM_010189.1	-4.8	

Glutathione peroxidase 1	*Gpx1*	NM_008160.1	-2.7	

Glutathione peroxidase 3	*Gpx3*	NM_008161.1	-2.0	

Glutathione S-transferase, mu 2	*Gstm2*	NM_008183.2	5.0	

Glutathione S-transferase omega 1	*Gsto1*	NM_010362.1	-2.1	

Guanine nucleotide binding protein (G protein), gamma 2 subunit	*Gng2*	NM_010315.2		2.9

Guanine nucleotide binding protein (G protein), gamma 10	*Gng10*	NM_025277		2.4

Hemochromatosis	*Hfe*	NM_010424.2	-5.3	

Immunoresponsive gene 1	*Irg1*	XM_127883	2.8	

Interferon, alpha-inducible protein 27	*2310061N23Rik*	NM_029803		3.9

Interferon induced transmembrane protein 2	*Ifitm2*	NM_030694.1	-2.0	

Interferon induced transmembrane protein 6	*Ifitm6*	XM_133956.3	-5.0	

Kruppel-like factor 6	*Copeb*	NM_011803.1		2.5

Lipocalin 2	*Lcn2*	NM_008491.1	-5.1	

LPS-induced TN factor	*Litaf*	NM_019980		2.6

Lymphocyte antigen 96	*Ly96*	NM_016923.1	-2.2	

Lysozyme	*Lyzs*	NM_017372	2.5	

Mannose receptor, C type 1	*Mrc1*	NM_008625.1	-7.6	

Membrane-associated ring finger (C3HC4) 8	*Mir*	NM_027920.3	-2.2	

Myeloperoxidase	*Mpo*	NM_010824.1	-3.3	

Peptidoglycan recognition protein 1	*Pglyrp1*	NM_009402.1	-2.5	

Peripheral myelin protein	*Pmp22*	NM_008885.1	3.5	

Peroxidasin homolog (Drosophila)	*2310075M15Rik*	XM_283052	2.0	

Peroxiredoxin 2	*Prdx2*	NM_011563.2	-3.5	

Peroxiredoxin 5	*Prdx5*	NM_012021		2.4

Phytoceramidase, alkaline	*Phca*	NM_025408.1	-2.1	

Prosaposin	*Psap*	NM_011179	-2.1	2.3

Regulator of G-protein signaling 1	*Rgs1*	NM_016846.2		4.8

S100 calcium binding protein A4	*S100a4*	NM_011311.1		4.6

S100 calcium binding protein A8 (calgranulin A)	*S100a8*	NM_013650.1	-4.4	

SAM domain and HD domain, 1	*Samhd1*	BC067198		2.8

Selenoprotein P, plasma, 1	*Sepp1*	NM_009155.3	-2.4	

Serum amyloid A 3	*Saa3*	NM_011315	4.5	

Solute carrier family 11 (proton-coupled divalent metal ion transporters), member 1	*Slc11a1*	NM_013612.1	-2.2	

Superoxide dismutase 2, mitochondrial	*Sod2*	NM_013671.2		2.9

Surfactant associated protein A1	*Sftpa1*	NM_023134.3	2.5	

Surfactant associated protein D	*Sftpd*	NM_009160.1	10.4	

				

**XII Genes mentioned in text but not categorized by Panther**				

Chemokine (C-X-C motif) ligand 12	*Cxcl12*	NM_013655.2	4.0	

Chemokine (C-X-C motif) ligand 13	*Cxcl13*	NM_018866.1	2.1	

Chemokine (C-X-C motif) ligand 16	*Cxcl16*	NM_023158		4.1

Expressed sequence AA467197	*AA467197*	NM_001004174.1		8.8

Lymphocyte antigen 6 complex, locus A	*Ly6a*	NM_010738.2	5.8	4.7

Immunity-related GTPase family, M	*Irgm*	NM_008326.1		2.8

Lymphocyte antigen 6 complex, locus C	*Ly6c*	NM_010741		3.0

Mus musculus lactotransferrin	*Ltf*	NM_08522.2	-16.8	

Proteasome (prosome, macropain) subunit, beta type 9	*Psmb9*	NM_013585.1		2.9

Proteasome (prosome, macropain) subunit, beta type 8	*Psmb8*	NM_010724		3.2

Proteasome (prosome, macropain) subunit, beta type 10	*Psmb10*	NM_013640.1		2.4

Serine (or cysteine) peptidase inhibitor, clade A, member 3G	*Serpina3g*	NM_009251.1		14.8

Three prime repair exonuclease 1	*Trex1*	NM_011637.4		2.0

Tryptophanyl-tRNA synthetase	*Wars*	NM_011710.2		2.2

Tumor necrosis factor (ligand) superfamily, member 13b	*Tnfsf13b*	NM_033622	3.1	3.0

Ubiquitin D (Ubd)	*Ubd*	NM_023137.2		4.2

Ubiquitin specific peptidase 18	*Usp18*	NM_011909.1		2.6

Expression was analyzed using Genome Studio and classified by immune function through Panther Immune Processes. Genes not classified by Panther but clearly related to immune processes were added manually (section XII). All processes were analyzed statistically with a p-value ≤ 0.05.

In contrast, of the 41 genes more highly expressed in lung i.n. samples, 8 are chemokines, namely *Xcl1*, *Cxcl1*, *Cxcl2*, *Ccl1*, *Ccl2*, *Ccl12*, *Ccl7 *and *Ccl8 *and a further three, *Oas12*, *Oas2 *and *Gbp1*, are interferon-stimulated genes (sections I and II Table 1). Chemokines such as *Xcl1*, *Ccl1*, *Ccl2*, *Ccl7 *and *Ccl8 *are responsible for recruitment of activated T cells into tissues (IUPHR database: http://www.iuphar-db.org/DATABASE/FamilyIntroductionForward?familyId=14). Because selection of genes in Panther is dependent on what has been curated, some genes that were differentially expressed and clearly related to immune processes but not classified by Panther, were added to Table 1 (section XII). Two other chemokine genes, *Cxcl12 *and *Cxcl13*, were preferentially expressed in lung i.n. samples (section XII Table 1), as was the T cell activation/memory marker *Ly6a*. Genes involved in lymphocyte effector function such as *Ifngr2 *(IFNγ receptor 2) and the TNF-stimulated gene *TNFsf13b *(BAFF) [16] were also upregulated in lung i.n.. These data suggests that Ad85A i.n. immunization establishes an activation program in lung CD8 T cells differing from that found in the spleen of Ad85A i.d. immunized mice.

It should be noted that some of the differences in gene expression shown in Table 1 are most likely due to contaminating cell types, which are a particular problem in the lung i.n. samples. For example, secretoglobin (*Scgbla1*) and surfactant associated protein D (*Spd*) show high fold-differences in the comparison but these proteins are predominantly produced by pulmonary Clara cells [17] or type II cells [18] and not T cells. Not surprisingly, these genes do not show differences in expression between lung i.n. and lung i.d. samples (below), which presumably have approximately equal contamination with non-lymphoid lung cells.

### Comparison of gene expression between lung i.n. and lung i.d

When lung i.n. (protective) were compared to lung i.d. (non-protective) CD8 T cells, we found a total of 245 differentially expressed genes (Additional file 2). Almost all the differentially expressed genes were more highly expressed in the lung i.n. samples. Gene Ontology analysis indicated that many of the highly expressed transcripts in the lung i.n. cells were related to immune processes (Figure 3B). Panther classification indicated that 86 of the 245 genes play a role in immune-related processes. Of these, 16 were classified as cytokine- and chemokine-mediated immunity and signaling genes, 11 as interferon-stimulated genes, 23 as T cell activation genes and 7 as apoptosis-related (Table 1). As before, a further 14 immunologically-related genes which were not curated by Panther were added to the table (section XII Table 1).

**Figure 4:**
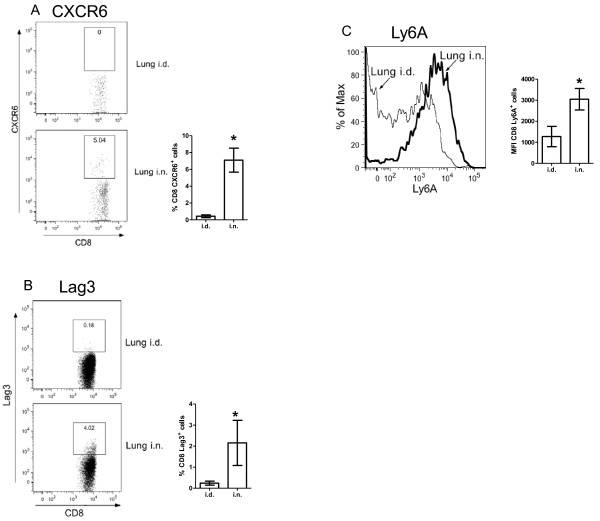
**CXCR6 expression on CD8 T cells from lungs of Ad85A immunized mice**. BALB/c mice were immunized i.d. or i.n. with Ad85A. At 3 weeks post-immunization, lung lymphocytes were isolated and the percentage of CD8 T cells expressing CXCR6 was determined by flow cytometry. (A) Representative FACS plots of CD8^+ ^gated cells showing CD8^+^CXCR6^+ ^cells in lungs of i.d. and i.n. immunized mice. The bar chart shows the mean ± SEM from 3 independent experiments with 3 mice per group. (B) Representative FACS plots of CD8^+ ^gated cells showing CD8^+^Lag3^+ ^cells in lungs of i.d. and i.n. immunized mice 8-12 weeks previously. The bar chart shows the mean ± SEM of 3 i.d. and 5 i.n. immunized mice. (C) A representative histogram showing the expression of Ly6a on lung CD8 T cells. The bar chart shows the mean fluorescence intensity (MFI) ± SEM of 3 i.d. and 5 i.n. immunized mice. * indicates p < 0.05 by Mann-Whitney test.

The lung i.n. sample expressed higher levels of the tissue-homing CC chemokines *Ccl1*, *Ccl2*, *Ccl3*, *Ccl4*, *Ccl5*, *Ccl7*, *Ccl8 *and CXC chemokines *Xcl1*, *Cxcl2*, *Cxcl9 *and *Cxcl16*, which are able to recruit resting and activated T cells as well as other immune cells. *Cxcr6*, which may aid in localization of activated T cells to non-lymphoid tissue [19-21], was also preferentially up-regulated in the lung i.n. samples. Flow cytometric analysis indicated that this was detectable on the surface of lung i.n. CD8 T cells but was present on very few CD8 T cells from lung i.d. immunized animals, confirming the differential expression detected by microarray analysis (Figure 4A). Similarly, increased expression of Lag3 protein was detected on the surface of a subset of lung i.n. CD8 T cells (Figure 4B). Measurement of chemokine production by *ex vivo *lung lymphocytes isolated from i.n. and i.d. immunized mice at 3 weeks post-immunization confirmed increased production of CCL2, CCL3, CCL4, CCL5, CXCL9 and CXCL16 (Table 2).

**Table 2:** Production of chemokines by lung lymphocytes *ex vivo *after immunization with Ad85A i.d. or i.n..

	Absorbance at 450nm (± SD)	
**Chemokine**	**i.d.**	**i.n.**

CCL2	0.89 (± 0.427)	1.368 (± 0.131)

CCL3	0.334 (± 0.066)	0.745 (± 0.008)

CCL4	0.278 (± 0.011)	0.760 (± 0.028)

CCL5	0.386 (± 0.098)	1.470 (± 0.211)

CXCL9	0.259 (± 0.059)	0.566 (± 0.034)

	Conc (pg/ml)	

CXCL16	3.34 (± 0.339)	6.095 (± 0.977)

Lung lymphocytes were isolated from mice immunized 3 weeks previously and cultured in RPMI+10% FCS for 6 hours at 37°C without antigen stimulation. The supernatants were assayed by ELISA for the indicated chemokines. The table shows the mean absorbance at 450 nm (± standard deviation (SD)) of 6 mice per group. For CXCL16, the mean concentration of CXCL16 (pg/ml, ± SD) of 6 mice per group is shown.

Compared to lung i.d. cells, lung i.n. CD8 T cells exhibited a strong anti-viral response, displaying higher levels of interferon-stimulated genes such as *Stat1, Oasl2*, *Gbp1 *and *Gbp2*. *Pdcd1lg*, *Ifit130*, *Wars *(tryptophanyl-tRNA synthetase) and diverse MHC Class II genes, indicating sustained activation of the IFNγ pathway. Additionally, high levels of activation markers such as *Ly6a*, *Ly6c *and *Ctla-4*, together with the antigen processing protease cathepsin S (*Ctss*), implied that there was a larger proportion of highly differentiated effector or memory CD8 T cells in the lung i.n. samples [22]. Increased expression of Ly6A on lung i.n. CD8 T cells was confirmed by flow cytometry (Figure 4C).

Serine peptidase inhibitor clade A member 3G, also known as *Serpina3g *or Spi2A exhibited the highest (14-fold) difference in expression in lung i.n. compared to lung i.d. samples. *Serpina3g *is highly expressed in effector and memory CD8 T cell populations [23]. During the contraction phase of immune responses, it protects expanded antigen-specific CD8 effectors from programmed cell death by inactivating lysosomal proteases [24], thereby regulating the contraction phase and ensuring that a high frequency of antigen-specific T cells remain to develop into memory cells [23]. The detection of high levels of *Serpina3g *transcript in lung i.n. samples suggests the presence of a pool of effector or memory T cells substantially larger than in the lung i.d. group, as confirmed by the frequency of antigen-specific cells (Figure 1).

Intriguingly, several inhibitory and activating molecules were also highly expressed in the lung i.n. samples, notably *Klrc1 *which is also known as NKG2A. NKG2A expression has been proposed as a marker for proliferative potential of CD8 memory T cells and may down-regulate effector activity as a means to limit immunopathology [25,26]. Interestingly another gene showing a high fold difference between lung i.n. and lung i.d. samples is *AA467197 *(*Nmes1*), which was recently reported to encode a microRNA and may act to dampen down excessive inflammation [27]. In contrast *Klrk1 *has been reported to function as a co-stimulatory receptor for activated CD8 T cells [28,29]. Along with *Serpina3g*, these genes share important roles in regulating CD8 effector function and survival.

Several MHC class II molecules showed higher expression in the lung i.n. samples. As it has been previously reported that surface expression of Class II is not detectable on mouse T cells [30], a possible explanation for the presence of the transcripts could be co-purification of CD8+ dendritic cells or expression of Class II on contaminating non-T cells. However, microarray datasets of mature and activated murine C57BL/6 CD8 T cells http://refdic.rcai.riken.jp/welcome.cgi, isolated to 99% purity by cell sorting, also show up-regulation of MHC Class II transcripts, suggesting that murine CD8 T cells can express MHC Class II mRNA.

In summary, the comparison of lung i.n. and lung i.d. samples demonstrated a striking relative increase in expression of chemokine genes involved in migration and retention of cells as well as in genes related to activation, regulation and survival of memory T cells.

### Comparison of gene expression between spleen i.n. and spleen i.d

As very few genes showed differential expression when spleen i.n. and i.d. samples were compared, we analyzed genes exhibiting fold differences ≥1.5 (Additional file 3). Even so, only 9 genes were differentially expressed and fold differences between only 1.5 and 2.8 were observed. Eight of the transcripts, *Klrc1*, *Gzmk *(granzyme K), *Ltf*, *Amy2*, *111001Rik*, *S100a6*, *Kcnk5 *(potassium channel subfamily k, member 5) and *Klrk1 *(killer cell lectin-like receptor, subfamily K, member 1) were higher in spleen i.d. samples, while the single transcript more highly expressed in spleen i.n. was *IGKV1-88_AJ231206_Ig_kappa_variable_1-88_289*, possibly an IgG kappa chain. Higher levels of transcripts of granzyme K, killer cell activators *Klrc1*, *Klrk1*, and the inflammation marker *S100a6 *suggested that spleen i.d. were more activated than spleen i.n. CD8 T cells. The few differences in gene expression between the splenic i.n. and i.d. samples may be because the antigen 85A-specific cells in the spleen represent a small proportion of the whole splenic CD8 population (Figure 1) or because the spleen, as a major hub of lymphoid traffic, may reflect irrelevant ongoing systemic immune responses at the time of sampling.

### Gene expression profile in the lungs after Ad85A i.n. immunization is distinct from common lung or *M. tuberculosis *responses

**Figure 5:**
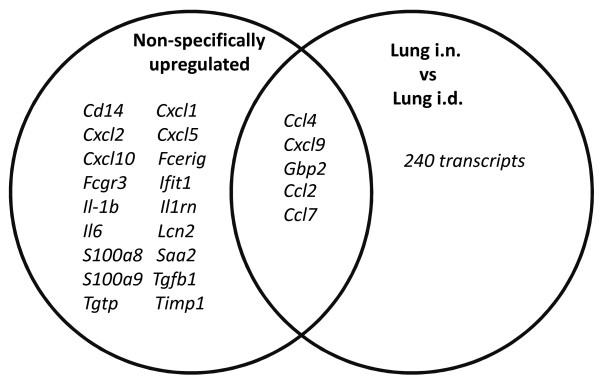
**The overlap of genes differentially expressed between lung i.n. and lung i.d., with genes reported to be related to a common lung inflammatory response**. Venn diagram showing the overlap between genes more highly expressed by lung i.n. than i.d. cells, with genes reported as upregulated in lung inflammation [31].

A common cluster of genes have been identified in mice and macaques as being non-specifically up-regulated during acute lung inflammation, irrespective of the type of stimulus. We compared the profile of differentially expressed genes between lung i.n. and lung i.d. samples with the genes which are subject to common up-regulation following a variety of inflammatory stimuli [31]. Of the 23 genes identified as most highly and commonly expressed following exposure to a range of pathogens and environmental insults, only 5 were shared with our lung i.n./lung i.d. differentially expressed gene set (Figure 5). These were *Ccl2*, *Ccl4*, *Ccl7*, *Cxcl9 *and *Gbp2*. A wider group of 50 genes is induced in response to pulmonary viral or bacterial infections [31]. Prominent among these are interferon-stimulated genes that are also more highly expressed in lung i.n. than lung i.d. samples, namely, *Aif1*, *Casp1*, *Ccl5*, *Ifit2*, *Ly6c*, *Psmb10*, *Psmb9*, *Psmb8*, *Stat1*, *Trex1*, *Ubd*, *Usp18 *and *Wars*. While the common responses described were measured during the acute phase of infection, less than 8 days post-exposure, i.n. administration of Ad85A induces expression of a subset of these acute-phase inflammatory molecules three weeks post-immunization, indicating that the expression profile induced may be a unique host response to Ad85A i.n. immunization.

Unsurprisingly, immunization with Ad85A induced an expression profile which was quite dissimilar to profiles generated during aerosol infection with *M. tuberculosis *or after immunization with BCG. Genes characteristically induced by mycobacteria, such as *Tlr2/4 *and *Indo *(IDO) [32], were not highly expressed in lung i.n. samples. However IFN-signalling pathway genes, such as *Icsbp1 *and *Stat1*, as well as genes associated with antiviral responses, such as *Oasl2*, *Gbp1 *and *Gbp2*, were strongly induced in lung i.n. samples, suggesting that virus was still present in the lungs at 3 weeks post-immunisation [5,7]. In spite of the differences in gene expression between mycobacterial and adenoviral immunization, several genes reported to be involved in *M. tuberculosis *clearance, including IFNγ and its receptor, *Il18*, *Cxcl13, Cxcl16*, *Cxcr6 *and *Serpina3g *are induced by lung i.n. immunization [32,33]. Thus the immune response to antigen 85A induced by i.n. immunization with Ad85A may be favourable for protection against subsequent pulmonary challenge with *M. tuberculosis*.

## Discussion

It is striking that the route of immunization with the Ad85A *M. tuberculosis *subunit vaccine is critical for protection against pulmonary *M. tuberculosis *challenge. Unlike conventional models of *M. tuberculosis *immunity, protection induced by Ad85A i.n. immunization in BALB/c mice is mediated by antigen-specific CD8 T cells in the lungs with a minimal contribution from CD4 T cells [7,13]. The antigen 85A-specific lung CD8 cells are maintained in a highly activated state by the continued presence of antigen [5] and are not dependent on recruitment from the periphery [7]. These cells inhibit *M. tuberculosis *growth early after pulmonary challenge [5]. In contrast, although mice immunized i.d. with Ad85A make a strong systemic CD8 response they are not protected against *M. tuberculosis*. Nonetheless splenic immune cells have been shown capable of inhibiting *M. tuberculosis *growth when transferred into the lungs [6]. These findings prompted us to determine whether differences in gene expression, determined by microarray analysis, might provide further insight into the protective mechanisms of lung and splenic CD8 T cells.

Comparison of lung i.n. versus spleen i.d. gene expression does not show a significant difference in the expression of *Ifng *or *Tnf *or other effector molecules, suggesting that lung and splenic CD8 T cells have equal potential to inhibit *M. tuberculosis *growth, as has been demonstrated by intra-tracheal transfer of splenic lymphocytes [6]. However, lung i.n. cells expressed higher levels of chemokines such as *Ccl1*, *Ccl2*, *Ccl7*, *Ccl8*, *Ccl12 *and *Xcl1 *and *Cxcl12*, suggesting that the main difference between the two populations is the presence of chemokines that enhance trafficking and retention of leukocytes in the lung. When lung i.n. and lung i.d. samples were compared, several additional chemokines were more highly expressed in lung i.n. samples, namely *Ccl3*, *Ccl4*, *Ccl5*, *Cxcl9 *and *Cxcl16*. Strikingly the only cognate chemokine receptor more highly expressed on lung i.n. than lung i.d. CD8 T cells is *Cxcr6*, the receptor for *Cxcl16*. *Cxcr6 *has been proposed to be a marker for retained lung T cells [34]. The presence of CXCR6 protein on a proportion of lung i.n. CD8 T cells was confirmed by flow cytometry (Figure 4).

Banchereau *et al. *reported that upon viral infection of humans, different subsets of chemokines are secreted in a temporally regulated manner, with *CXCL1*, *CXCL2*, *CXCL3 *and *CXCL16 *being secreted first to allow homing of naïve T cells, then *CCL3*, *CCL4*, *CCL5*, *CXCL8*, *CXCL9*, *CXCL10 *and *CXCL11 *to sustain localization of activated T cells, and finally *CCL19*, *CCL22 *and *CXCL13 *[35]. In our model, some chemokines reported to be part of the "1^st ^and 2^nd ^waves" of chemokine expression by DC cells, specifically *Xcl1*, *Cxcl9*, *Cxcl16*, *Ccl1*, *Ccl2*, *Ccl3*, *Ccl4*, *Ccl5*, *Ccl7 *and *Ccl8 *are expressed at increased levels in lung i.n. samples at 3 weeks post-infection. A possible explanation for the presence of transcripts involved in activation and recruitment of T cells at this late time point in lung i.n. and not lung i.d. samples is the persistence of Ad85A in the lung [5] inducing sustained expression of IFNγ and STAT1 which are able to coordinate expression of numerous members of the chemokine family [36]. Additionally, synergism between IFNγ and TNF, which are produced by 85A-specific T cells (Figure 1), may lead to upregulation of a further subset of genes involved in T cell activation and recruitment, including *Irg1*, MHC Class II molecules and the chemokine genes including *Cxcl9 *[37]. Sustained expression of these chemokines may recruit and retain CD8 T cells in the lung so that they are able to control *M. tuberculosis *as soon as the mycobacteria are present in the lung [5]. In addition, these chemokines may also recruit other activated immune cells to the lung, such as macrophages, neutrophils and NK cells, ensuring that they are present *in situ *at the time of infection. Since a hallmark of *M. tuberculosis *infection is the very slow initiation of immune responses and delayed migration of T cells to the lung [38,39], retention of immune T cells in the lungs, as induced by persistent antigen stimulation and consequent chemokine production, may be an important reason for the efficacy of i.n. immunization with Ad85A [5,38,40,41].

## Conclusions

Our microarray analysis represents the first *ex vivo *study comparing gene expression profiles of CD8 T cells isolated from distinct sites after immunization with an adenoviral vector by different routes. It confirms earlier phenotypic data indicating that lung i.n. cells are more activated [5] than lung i.d. CD8 T cells. Thus it appears that the state of activation of the lung i.n. CD8 T cells is critical for their ability to inhibit *M. tuberculosis *growth early after infection. Lung i.n. cells also highly express many chemokines as well as the CXCR6 receptor. We suggest that continued expression of these molecules, as a consequence of the persistence of antigen 85A, helps to retain the cells in the lungs. These two properties may explain why this immunization regime is effective. An intriguing question for the future is whether the presence of a population of highly activated CD8 T cells in the lungs is hazardous for the host. However, lung i.n. samples co-express both genes activating and regulating inflammation, suggesting that the lungs may be in a stable well-balanced state.

## Competing interests

The authors declare that they have no competing interests.

## Authors' contributions

LNL performed the RNA isolation experiments, data analysis of the microarray results and drafted the paper; DB performed the microarray analysis and statistical analysis of the data; EZT and EOR performed the intracellular cytokine analysis and *M. tuberculosis *challenge studies; JR directed the microarray studies; EZT and PCLB conceived the study design, directed the data analysis and drafted the manuscript. All authors read and approved the final manuscript.

## Pre-publication history

The pre-publication history for this paper can be accessed here:

http://www.biomedcentral.com/1755-8794/3/46/prepub

## Supplementary Material

Additional file 1 List of 550 transcripts differentially expressed between lung i.n. and spleen i.d. Click here for file

Additional file 2 List of 245 transcripts differentially expressed between lung i.n. and lung i.d. Click here for file

Additional file 3 List of 9 transcripts differentially expressed between spleen i.n. and spleen i.d. Click here for file
